# Editorial: New Insights into the Complexity of Tumor Immunology in B-cell Malignancies: Tumor Immunology and Immunotherapy

**DOI:** 10.3389/fonc.2022.853620

**Published:** 2022-02-03

**Authors:** Martina Seiffert, Etienne Moussay, Jérôme Paggetti

**Affiliations:** ^1^ Division of Molecular Genetics, Molecular Genetics, German Cancer Research Center (DKFZ), Heidelberg, Germany; ^2^ Tumor-Stroma Interactions, Department of Cancer Research, Luxembourg Institute of Health, Luxembourg, Luxembourg

**Keywords:** leukemia, lymphoma, tumor microenvironment, stromal cells, chronic lymphocytic leukemia (CLL), immunotherapy, anti-tumor immunity

With the recent advances in cancer immunotherapy, great expectations have been raised for the successful implementation of these novel treatment concepts for patients with B-cell malignancies. The malignant cells in these cancer entities are in constant interaction with non-malignant immune cells and stromal cells in primary and secondary lymphatic tissues, and many B-cell malignancies are associated with immune defects. Even though treatment with immune checkpoint inhibitors or CAR T-cell therapies showed efficacy in some patients, the majority did not respond, for so far unclear reasons. To improve response rates, a better understanding of the cross-talk between the cancer cells and their microenvironment, and of immune escape and treatment resistance mechanisms are urgently needed. The collection of reviews, opinion, method, and original research articles in this Research Topic focuses on the complexity of the tumor microenvironment (TME) in B-cell malignancies and on novel concepts of immunotherapy for patients with these deadly diseases.

In a detailed and timely review, Apollonio et al. summarize current knowledge on tumor intrinsic and extrinsic mechanisms critical to anti-tumor immune responses, as well as sensitivity to immunotherapies in B-cell lymphomas. They discuss the current understanding concerning the role of T cells and inflammatory signaling in these processes. The article further focuses on co-evolving stromal cells and their regulation of immune responses within the TME.

Similarly, Delahaye et al. discuss in a mini review article the current knowledge about protective niches in B-cell acute lymphoblastic leukemia (B-ALL) and the development of therapies targeting the crosstalk between leukemic cells and their microenvironment.

To improve immunotherapy approaches for patients with chronic lymphocytic leukemia (CLL), a better understanding of their defective immune system is required. Griggio et al. focus in their informative review on the main immune defects affecting patients with CLL, also describing the complex networks leading to immune evasion and tumor progression. They further summarize the evolution of immune-based therapeutic approaches, including immunomodulatory drugs, monoclonal antibodies, and immunotherapeutic strategies aiming at activating or administering leukemia-reactive immune effector cells.

Considering the recent advances in our understanding of T-cell activity and defects in CLL, a timely review by Vlachonikola et al. focuses on the binary, contradicting role of T lymphocytes in CLL. They describe the imbalanced composition of the T-cell compartment in CLL and how it is driven by antigen selection. Further, they focus on T-cell defects and perspectives of novel immunotherapy approaches to overcome them.

One of the main immune defects described for patients with CLL, is the failure to develop proper immune synapses between malignant B cells and effector T cells which was linked to altered cytoskeleton properties. In an original research article, Wurzer et al. analysed immune synapse formation between CLL and NK cells and suggest a novel immune escape mechanism in CLL. They show that fast actin remodeling in CLL cells upon contact with NK cells prevents their effective NK-cell-mediated killing. These results highlight the critical role of the actin cytoskeleton in CLL cells in driving resistance against NK cell cytotoxicity and provide a new potential therapeutic point of intervention to target CLL immune escape.

Due to the increasing interest in the TME, extensive analyses revealed a tremendous complexity and heterogeneity of the cellular microenvironment in cancer. The development of novel technologies for single-cell analysis, like mass cytometry, enhances the number of cellular features that can be surveyed simultaneously and allows for resolving complex cellular systems. In a detailed method article by Gonder et al., the use of mass cytometry for a better characterization of cells in the TME of CLL is described. This includes a protocol for antibody panel design and validation, sample preparation and acquisition, machine set-up, quality control, and analysis of cells. Additionally, advantages and pitfalls of this technique are discussed.

Richter Syndrome (RS) is a highly aggressive B-cell malignancy that arises due to the transformation of CLL, and the development of effective treatment options for RS patients is an urgent clinical need. In a very informative review article, Augé et al. provide an extensive overview of genomic aberrations associated with RS. The article further focuses on the role of the PD-1/PD-L1 immune checkpoint axis in RS and discusses the potential of targeting this checkpoint as novel therapeutic option for patients with this aggressive disease.

Multiple myeloma (MM) is an aggressive malignancy of plasma cells associated with pathological changes in the bone marrow microenvironment. Due to these, the majority of patients with MM develop anemia. Immunomodulatory agents like pomalidomide or lenalidomide are used for the treatment of MM and associated with the recovery from anemia. Verma discusses in an interesting opinion article the potential mode-of-action underlying this observation.

Inhibitors of phosphoinositide 3-kinase (PI3K) are approved for the treatment of CLL and other B-cell lymphoma. But the PI3K signaling pathway is not only limited to cancer cells but also crucial for many components of the TME which has to be considered when treating patients with PI3K inhibitors. Aydin et al. address this important issue in their detailed review article. They summarize published data that show an impact of PI3K inhibition on the TME with a specific focus on CLL, and discuss how a better understanding of these effects of PI3K inhibitor-based therapies can serve as a rationale for the development of improved drugs or of novel combinatory treatment strategies in CLL.

Novel immunotherapies, like immune checkpoint blockade or CAR T-cell therapy showed efficacy in some patients with B-cell lymphoma, but for the majority of cases, this treatment approach failed. As a potential resistance mechanisms in Hodgkin and Non-Hodgkin lymphoma, Albakova et al. explore in their interesting review article the involvement of heat shock proteins (HSPs). As HSPs are highly expressed in lymphoma cells and are known to modulate immune responses and inhibit apoptosis, a better understanding of their role in anti-tumor responses may help in the development of more effective immunotherapy in B-cell lymphoma.

Activation of Toll-like receptor (TLR) signaling *via* synthetic agonists is an immunomodulatory approach currently tested in several clinical trials for cancer. In an original research article, Lu et al. show that treatment of established A20 B-cell lymphoma in mice with a synthetic TLR4 agonist controlled tumor development. This effect was associated with T-cell inflammation and dependent on CD8^+^ T cells. Interestingly, the treatment response depended on TLR4 expression in B-cell lymphoma cells. TLR4 agonist treatment of lymphoma cells led to their enhanced antigen-presentation and increased their apoptosis. It was further sufficient to induce protective CD8^+^ T-cell responses. Therefore, the therapeutic potential of TLR4 activation in B-cell lymphoma should be further explored.

A novel interesting treatment approach for primary central nervous system lymphomas (PCNSL) harboring B-cell receptors that recognize the common auto-antigens neurabin-I and SAMD14 is presented in the original research article by Bewarder et al. They generated a full-length IgG1 or Fab antibody format containing the PCNSL-reactive epitope of SAMD14/neurabin-I and showed specific binding of these constructs to lymphoma cells as well as their effective killing in coculture with PBMC.

Altogether, the collection of articles in this Research Topic provides a comprehensive overview of the immune microenvironment in B-cell malignancies and the potential of new immunotherapy approaches for these diseases, thereby supporting the development of improved treatment regimens for the patients.

## Author Contributions

All authors edited the Research Topic. MS wrote the editorial. All authors contributed to the article and approved the submitted version.

## Funding

This work was supported by grants from the Fonds National de la Recherche Luxembourg to JP and EM (C20/BM/14592342 and C20/BM/14582635).

## Conflict of Interest

The authors declare that the research was conducted in the absence of any commercial or financial relationships that could be construed as a potential conflict of interest.

## Publisher’s Note

All claims expressed in this article are solely those of the authors and do not necessarily represent those of their affiliated organizations, or those of the publisher, the editors and the reviewers. Any product that may be evaluated in this article, or claim that may be made by its manufacturer, is not guaranteed or endorsed by the publisher.

